# Clinical and neuroradiological findings in patients with Anti-AMPAR encephalitis: associations with outcomes

**DOI:** 10.1186/s12883-025-04608-4

**Published:** 2026-01-02

**Authors:** Shu Jiang, Chao Zhang, Xinyi Wang, Peng Zhang

**Affiliations:** 1 Department of Radiology, The First Affiliated Hospital of Shandong First Medical University & Shandong Provincial Qianfoshan Hospital, Jinan, 250014 Shandong Province China; 2 Department of neurology, The First Affiliated Hospital of Shandong First Medical University & Shandong Provincial Qianfoshan Hospital, Jinan, 250014 Shandong Province China

**Keywords:** Anti-alpha-amino-3-hydroxy-5-methyl-4-isoxazolepropionic acid receptor, Limbic encephalitis, Brain MRI, Outcome

## Abstract

**Background:**

Encephalitis associated with antibodies against the α-Amino-3-Hydroxy-5-Methyl-4-Isoxazolepropionic acid receptor (AMPAR) is an extremely rare type of antibody-mediated encephalitis. Its clinical phenotype and neuroradiological characteristics remain incompletely described.

**Methods:**

We present four cases of anti-AMPAR encephalitis, and a comprehensive literature review highlighted the diverse clinical experiences, with specific attention to the clinical and radiographic characteristics of anti-AMPAR encephalitis, as well as the association between clinical, neuroradiological presentations and outcomes.

**Results:**

A total of 89 patients with anti-AMPAR encephalitis were included in this review. Clinical presentations at the onset of anti-AMPAR encephalitis were diverse, including behavioral, cognitive, motor, and sensory manifestations. Neuroradiological findings cannot be restricted to the limbic system. They may spread to unexpected sites, like basal ganglia, cerebellum, cerebral cortex regions, and even diffuse hyperintensities, or patient may have completely normal brain magnetic resonance imaging (MRI). Fisher’s exact test showed that there was non-significant difference in the clinical symptoms and outcomes among different MRI presentations.Logistic regression analysis showed no significant correlations between clinical, neuroradiological findings and unfavorable outcome.

**Conclusion:**

Anti-AMPAR encephalitis mainly presents as limbic encephalitis, and most of the patients had positive brain MRI results. The range of hyperintensity on MRI may not be directly associated with patients’ clinical symptoms and outcomes. For the expanding clinical profile of encephalitis associated with antibodies against AMPAR, careful databasing of new cases will facilitate more definitive study in the future.

## Introduction

Autoimmune encephalitis is a group of immune-mediated inflammatory diseases affecting the nervous system, primarily characterized by antibodies targeting directly at cell-surface antigens of neurons and induce varieties of neuropsychiatric disturbances [1, 2]. First described by Lai et al. [3] in 2009, encephalitis associated with anti-α-Amino-3-Hydroxy-5-Methyl-4-Isoxazolepropionic acid receptor (AMPAR) represents a subtype of autoimmune encephalitis linked to anti-neuronal surface antigens. As ionotropic glutamate receptors (GluR), AMPARs assemble into tetramers consisting of GluR1, GluR2, GluR3, and GluR4 subunits, with the GluR2 subunit showing enriched distribution in the hippocampus, amygdala, and cerebral cortex [3, 4]. AMPARs mediate fast synaptic transmission in the central nervous system and are essential for synaptic plasticity, learning, and memory. Patients typically present with diverse symptoms such as confusion, amnesia, seizures, encephalopathy, and psychosis [5, 6].

To date, fewer than 100 cases of anti-AMPAR encephalitis have been described in the published literature, presenting substantial diagnostic hurdles owing to the condition’s clinical heterogeneity and low incidence [7–13]. Magnetic resonance imaging (MRI) frequently demonstrates abnormalities with a stereotyped distribution, including a distinct predilection for the bilateral temporal lobes [14] In the present study, we report four cases of anti-AMPAR encephalitis, describe the corresponding clinical and neuroradiological findings, discuss the clinical courses and outcomes, and review the existing literature on brain MRI and clinical features of this disease. Detailed characterization of the clinical presentation, neuroradiological findings, and outcomes of anti-AMPAR encephalitis may facilitate improved recognition of patients with suspected AMPAR-associated encephalitis.

## Materials and methods

### Clinical cases

By retrospectively reviewing patients with positive AMPAR antibodies in CSF or serum, a total of 4 patients diagnosed with anti-AMPAR encephalitis were enrolled in this study between April 2021 and November 2023. Demographic data, clinical manifestations, laboratory findings (including CSF analysis, anti-neuronal antibodies in serum/CSF), scalp electroencephalography (EEG), brain MRI, and oncological screening results, as well as treatment regimens and therapeutic responses, were extracted from the patients’ outpatient and inpatient medical records. Written informed consent was obtained from each patient. This study was approved by the Research Ethics Committee of The First Affiliated Hospital of Shandong First Medical University.

### Systematic review and data extraction

**Fig. 1:**
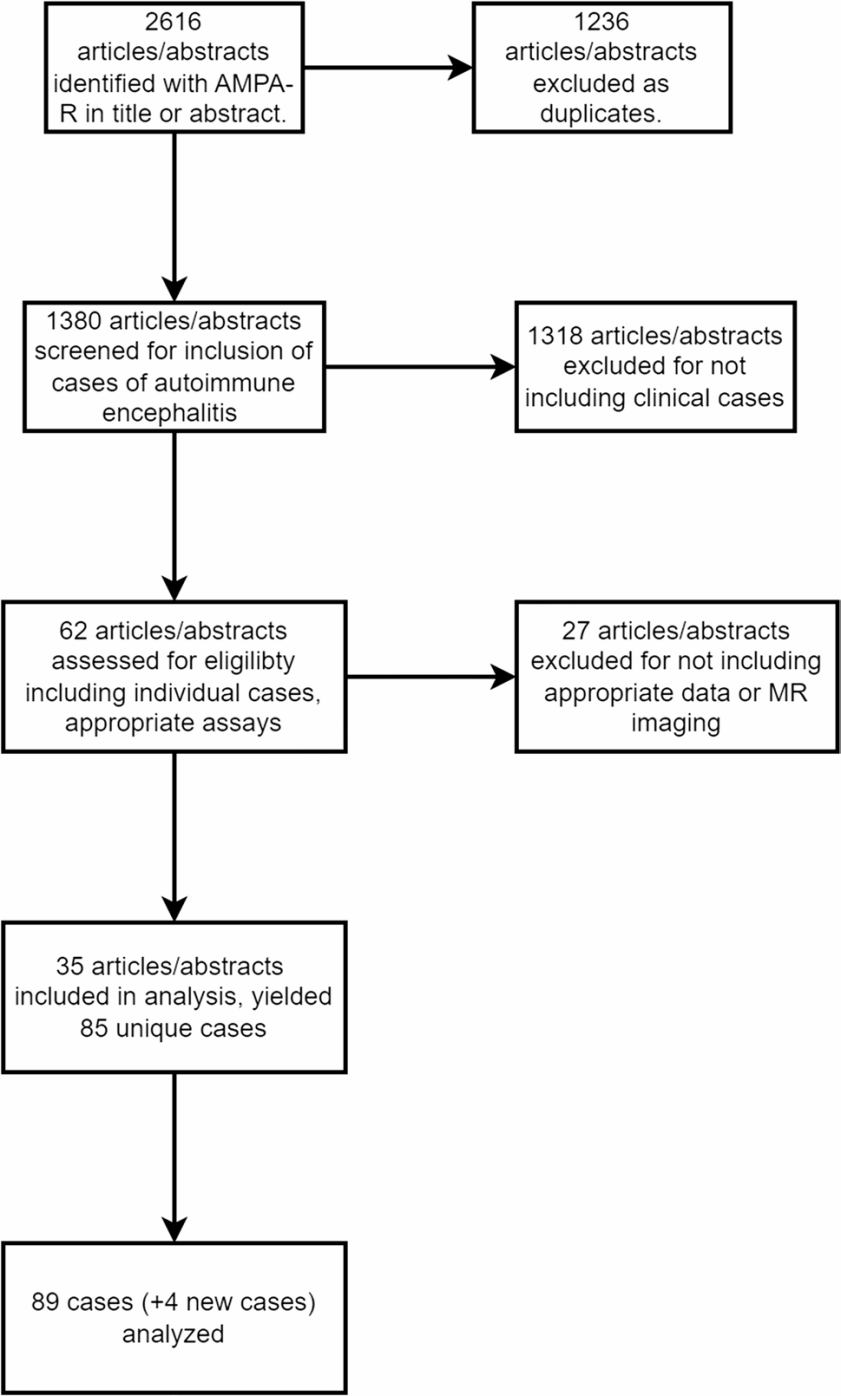
PRISMA diagram. PRISMA diagram summarizing manucript selection from systematic literature review

To identify relevant literature on anti-AMPAR encephalitis, we conducted a systematic search of the PubMed database using the key terms “autoimmune encephalitis” and “AMPAR antibodies”. This search was repeated periodically to ensure comprehensiveness, with the final search conducted on May 25, 2025. The inclusion criteria were as follows: (1) the patient diagnosed with anti-AMPAR encephalitis; (2) the presence of AMPAR antibodies in the cerebrospinal fluid or serum antibody test; (3) including MRI data. Patients with other clearly defined neurological diseases were excluded. Abstracts of the retrieved records were screened for eligibility, and a total of 62 manuscripts were selected for full-text review. Twenty-seven manuscripts were excluded: these either did not report unique cases of anti-AMPAR encephalitis or focused primarily on other disease entities. Data were subsequently extracted from the unique cases documented in the remaining 35 manuscripts. The PRISMA diagram was shown in Fig. 1.

### Data analysis

Abnormal findings on brain MRI were extracted and categorized based on the cortical anatomical regions as defined in Freesurfer [15]. T2-weighted hyperintensity was the most frequently observed lesion type.

All analyses were conducted using the statistical software of SPSS (version 19.0; IBM). The associations between different MRI presentations and clinical symptoms, outcomes were quantified using Fisher’s exact test. The relationship between demographic, clinical variables and neuroradiological presentation on outcomes were quantified using logistic regression. Death and partial recovery were defined as unfavorable outcome, and return to baseline was defined as favorable outcome. Statistical significance was defined as *p* < 0.05.

## Result

We identified 4 patients with anti-AMPAR encephalitis, including 2 women and 2 men. Their median age was 56 years (range 32–57 years). All patients were positive for AMPAR antibodies in both serum and CSF samples (antibody titer range, 1:10 to greater than 1:320). In 3 of the 4 patients, the clinical diagnosis of limbic encephalitis was confirmed by the MRI findings of mesiotemporal increased fluid-attenuated inversion recovery (FLAIR)/T2WI signal abnormalities. In patient 3, the brain MRI was normal. During the follow-up period ranging from 75 to 131 weeks, no tumor state was observed in our patients. The main clinical data of our 4 cases are reported in Table 1.

**Table 1:** Clinical features and outcomes in 4 cases with anti-AMPAR encephalitis

Case	Age, y/sex	Clinical presention	Other symptoms during course of the disease	EEG	Brain MRI	CSF	CSF titer	Serum titer	Tumor state	Treatment	Outcomes at follow-up
1	32/F	Memory loss, limb weakness	Perioral numbness, slow response, fever	slow-wave activity	Bilateral temporal lobe	Normal	1:100	1:100	Not found	IVIG, steroids	Progressive improvement, persistence of significant cognitive impairment
2	52/F	Memory loss, limb weakness	Fever, weakness of limbs, slow reaction	slow-wave activity	Left frontal parietal lobe, bilateral hippocampal and temporal lobe	Normal	1:100	1:100	Not found	IVIG, steroids	No significant improvement
3	57/M	Memory loss	(-)	Normal	Normal	Normal	1:100	1:10	Not found	Steroids	Progressive improvement
4	53/M	Confusion, memory loss	Slow response	NA	Bilateral hippocampal and temporal lobe	WBC elevated	1:320	1:320	Not found	IVIG, steroids	Progressive improvement

Abbreviations: *MRI* Magnetic resonance imaging, *AMPAR* α-Amino-3-Hydroxy − 5-Methyl-4-Isoxazolepropionic acid receptor, *EEG* Electroencephalogram, *CSF* Cerebrospinal fluid, *IVIG* Intravenous immunoglobulin, *WBC* White blood cell, *NA* Not available

## Case reports

### Patient no. 1

A 32-year-old woman presented to the hospital with perioral numbness accompanied by limb weakness and sleep disorders for more than 10 days. The patient gradually showed memory loss, slow reaction, and reduced speech. Neurological examination showed that the reaction was slow, the comprehension and calculation power decreased, and negative Babinski signs as well as negative meningeal irritation signs.

**Fig. 2:**
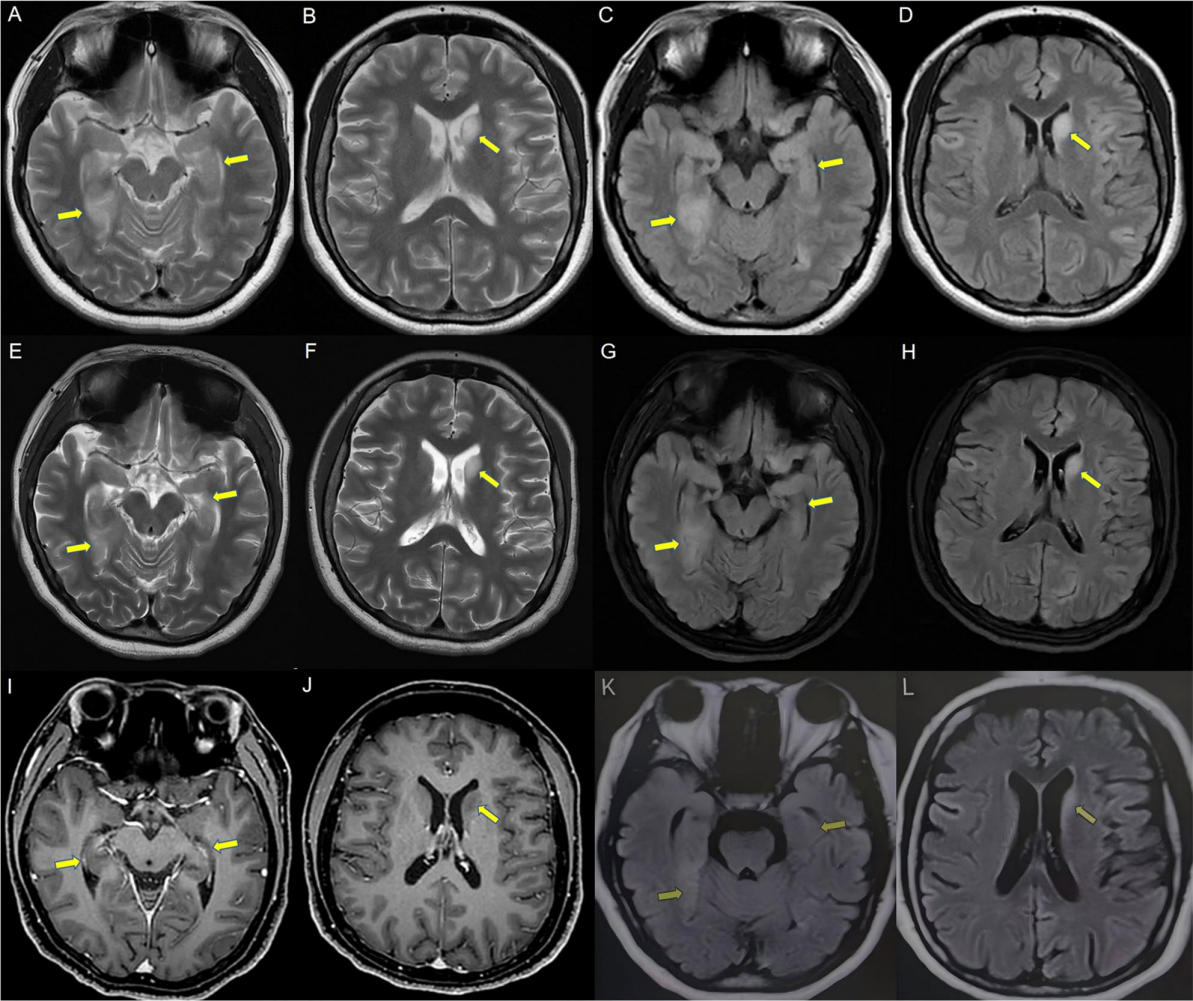
Brain MRI findings of patient No.1. **A** ~ **D** After symptom onset, increased T2WI and T2-fluid-attenuated inversion recovery (FLAIR) signal could be observed in bilaterally medial temporal lobe and hippocampus, right caput nuclei caudati. **E** ~ **J** Follow-up imaging 7 days later, the range of the T2WI and T2-FLAIR hyperintensity decreased slightly. Contrast image showed no enhancement. **K**, **L** Follow-up imaging 20 days later, the range of hyperintensity decreased significantly

After admission, the blood test showed an increase in white blood cells and neutrophils. And other routine serum tests were normal. Routine CSF tests exhibited normal results. However, the EEG showed increased slow-wave activity during the waking period and no typical epileptic discharge. Brain MRI revealed increased T2WI and T2-FLAIR signal involving bilaterally medial temporal lobe and hippocampus, right caput nuclei caudate (Fig. 2A ~ D). PET-CT indicated circular nodules in the mediastinum with a slight increase in metabolism and a high possibility of benign lesions. AMPAR antibodies were both detected in CSF(1:100) and serum(1:100).

Methylprednisolone pulse therapy and intravenous immunoglobulin (IVIG) were started. MRI revealed that scattered T2WI and T2-FLAIR hyperintensity had decreased compared with 7 days (Fig. 2E ~ J) after onset. However, the symptoms of the patient did not improve, and then further aggravated, almost unable to speak, unable to walk, and the right upper limb involuntary movement. At the follow-up 20 days later, this patient demonstrated an improvement in symptoms but still had persistent cognitive impairment (mainly including memory deficits and executive dysfunction), while the range of hyperintensity decreased significantly in brain MRI (Fig. 2K, J).

### Patient no. 2

**Fig. 3:**
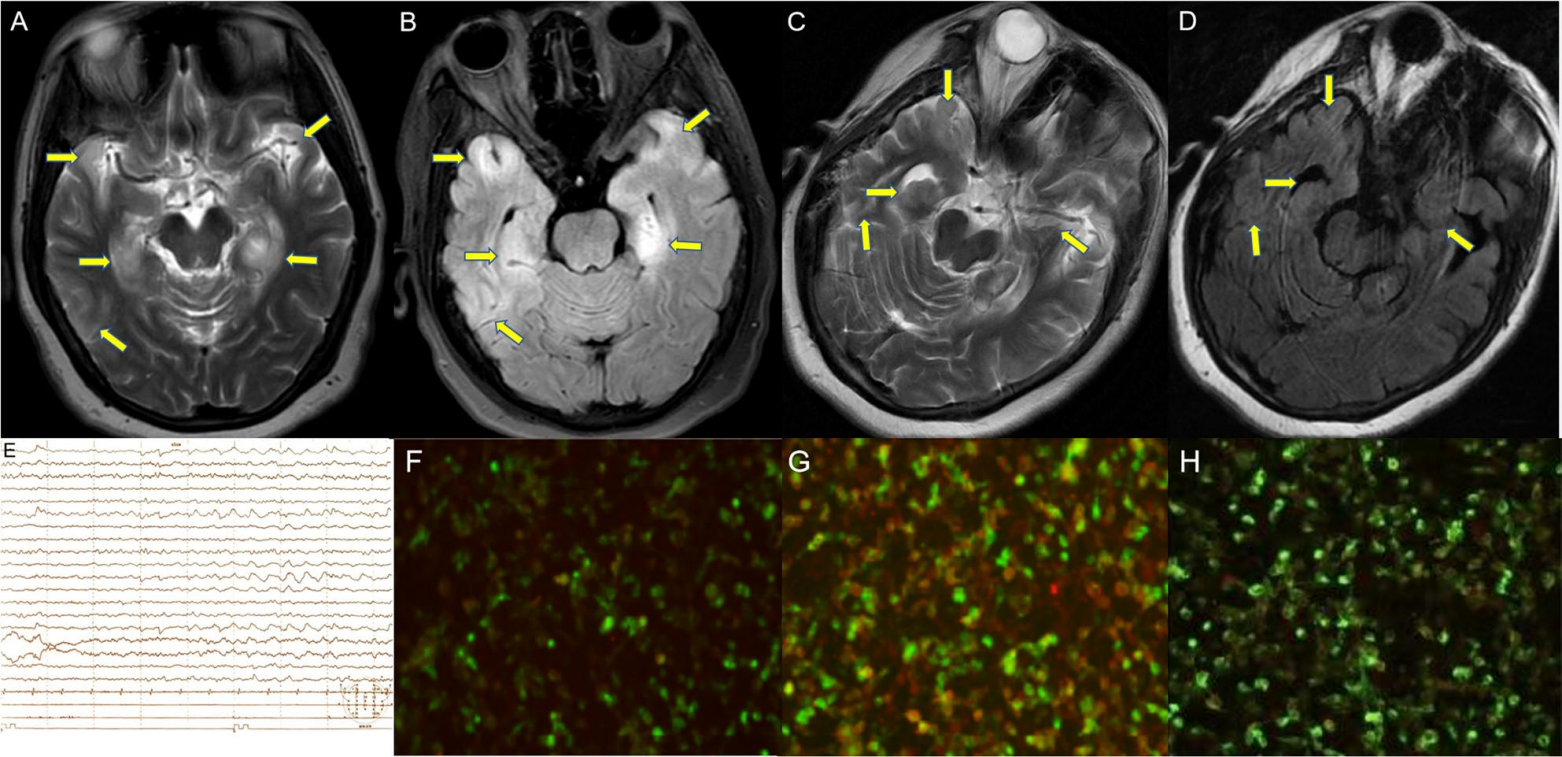
Brain MRI and clinical findings of patient No.2. Brain MRI showing increased scattered T2WI and T2-FLAIR hyperintensity in bilateral temporal lobe and hippocampus (**A**, **B**) and 42 days (**C,** **D**) after onset, the range of hyperintensity decreased significantly. (**E**) EEG showed slow-wave activity in right hemisphere of the brain background slowing suggestive of diffuse encephalopathy. (**F**, **G**) Presence of autoimmune antibodies in serum and CSF. (H) At 42 days after onset, the titer of antibodies in the serum was decreased

The patient, a 52-year-old woman, was admitted to the hospital because of memory loss, accompanied by fever, weakness of limbs, slow reaction, dull expression, and decreased sleep. No clear positive findings were observed in the physical examination except for decreased muscular strength in the right lower limb. Blood routine, biochemical, thyroid function tests, and routine CSF were all normal. Brain MRI showed increased scattered T2WI and T2-FLAIR hyperintensity in the bilateral temporal lobe and hippocampus (Fig. 3A, B). The slow-wave activity was observed in the right hemisphere of the brain in the background of the EEG examination (Fig. 3E). PET-CT did not detect any tumors. AMPAR antibody was positive in both serum and CSF (the titer of serum and CSF were both 1:100) (Fig. 3F, G).

Methylprednisolone pulse therapy (500 mg/day for 5 days) was commenced, and then IVIG was added to the therapeutic regime. The patient was poorly responsive to treatment. The patient remained unconscious, silent, expressionless, weak in both lower limbs, and unable to walk. After 42 days, the range of hyperintensity decreased significantly (Fig. 3C, D) while reexamination of AMPAR antibody showed the level in serum remained 1:100 (Fig. 3H), respectively, and the condition was not improved.

### Patient no. 3

A 57-year-old man was admitted to our hospital, presenting with anterograde memory loss for a month His memory disturbances, particularly short term memory loss and disorientation, had aggravated gradually. Neurological examination showed no obvious abnormality. Normal investigations included blood tests, liver and renal function tests, antinuclear antibodies, EEG, and blood cultures. HIV and Treponema pallidum serology were negative. CT and MRI brain were both normal. Oncologic screening was negative. AMPAR antibody was positive in both serum and CSF (the titer of serum was 1:10 and CSF was 1:100).

Methylprednisolone pulse therapy was started. The symptom of memory loss has no significant improvement. After discharge, the memory decline was gradually improved.

### Patient no. 4

The patient, a 53-year-old male, appeared perinasal herpes 10 days ago, and then gradually developed trance and memory decline, slow response and reduced voluntary speech. Neurological examination showed the higher nerve function was decreased and Kernig’s sign is suspiciously positive. Blood routine, biochemical, and thyroid function tests were all normal. Routine CSF analysis showed an elevated white blood cell count (87 × 10^6^/L, 90% monocytes) and others were normal. MRI showed scattered hyperintensity of the bilateral hippocampus and temporal lobes on T2WI and T2-FLAIR sequences. AMPAR antibody was positive in both serum and CSF (the titer of serum and CSF were both 1:320).

Methylprednisolone pulse therapy and IVIG were started. After 2 weeks of treatment, the effect was not obvious and the patient developed respiratory failure. The patient was transferred to the ICU for treatment. IVIG and rituximab treatment was given again. After treatment, the patient’s consciousness was improved, but still can not speak. At the last follow-up, cognitive function was improved, and no abnormal altered mental status, dystonia, seizures, and autonomic dysfunction were noted.

### Systematic literature review

To evaluate the clinical presentations, outcomes and neuroradiological characteristics of anti-AMPAR encephalitis, we reviewed the previously reported studies. A systematic review of the literature revealed a total of 89 patients with anti-AMPAR encephalitis and neurological involvement.

Demographic features and clinically relevant symptoms and signs are presented in Table 2. 66% (59 of 89) of the cases occurred in women, and the median patient age was 51.5 years (range, 12–92 years). Clinical presentations at the onset of anti-AMPAR encephalitis were diverse, characterized by the presence or absence of five symptoms: confusion (31 patients), limbic encephalitis (45 patients), amnesia (47 patients), seizures (18 patients), and psychiatric disturbances (27 patients). We acknowledge the potential for overlap between terminology “limbic encephalitis” (describing altered level of consciousness, seizures and psychoses) and confusion. Other clinical symptoms and signs were reported sporadically, including fever, focal weakness, dysphagia, sleep disorders, ataxia, involuntary movements, autonomic dysfunction, aphasia, sensory symptoms. Cancer was found in 40 of 89 patients (44.9%) with known cancer status, most commonly lung carcinoma and thymoma (16 thymoma, 12 lung cancers, 5 breast cancers, 4 ovary cancers, 1 thyroid cancer, 1 osteosarcoma, 1 bladder carcinoma). Treatment of anti-AMPAR encephalitis includes immunotherapy and oncological treatment if tumors are comorbid. Immunotherapy is composed of first-line therapies (IVIG, steroids, and plasmapheresis). In general, outcomes were favorable. Ten patients with anti-AMPAR encephalitis died (11.2%), most commonly of complications related to underlying malignancy. 34 patients returned to baseline and 45 patients remained partial impairment.

**Table 2:** Demographic features and clinically relevant symptoms

Variables	Range	Mean
Sex		30 M/59 F
Age (years)	12–92	51.5
**Clinical symptoms**	N	% positive
Limbic encephalitis	45	50.6
Confusion	31	34.8
Amnesia	47	52.8
Seizures	18	20.2
Psychiatric complaints	27	30.3
**Clinical studies**	N	% positive
Tumor identified	40	44.9
Thymoma	16	
Lung	12	
Breast	5	
Ovary	4	
Thyroid	1	
Osteosarcoma	1	
Bladder carcinoma	1	
MRI abnormal	66	74.2
Treatment	87 available	
Steroids	11	
IVIG	8	
Steroids + IVIG(+ plasmapheresis)	41 (32 + 9)	
Steroids + plasmapheresis	3	
Tumor treatment(+ IVIG)	8 (3 + 5)	
Tumor treatment + steroids	5	
Tumor treatment + steroids + IVIG	8	
Tumor treatment + steroids + IVIG + plasmapheresis	3	

*Abbreviations*: *MRI* Magnetic resonance imaging, *IVIG* Intravenous immunoglobulin

Up to 74.2% of the patients had abnormal brain MRI with a stereotyped topography including a clear predilection for bilateral temporal lobes. However, it should be noted that patients with anti-AMPAR encephalitis may have completely normal brain MRI and the imaging abnormalities may spread to unexpected sites, like basal ganglia, cerebellum, cerebral cortex regions, and even diffuse hyperintensities. We classified 89 cases into three groups of normal group (*n* = 23), limbic system hyperintensities group (*n* = 43) and non-limbic system hyperintensities group (*n* = 23) according to the MRI presentations. Limbic system hyperintensities defined as T2/T2-FLAIR hyperintensities in limbic system and non- limbic system hyperintensities involved structures like basal ganglia, cerebellum, cerebral cortex regions, and even diffuse hyperintensities. Fisher’s exact test was performed to determine if the clinical symptoms and outcomes of anti-AMPAR encephalitis depended on the MRI presentations (Table 3). The results showed that there was non-significant difference in the clinical symptoms and outcomes among different MRI presentations. To determine the clinical and neuroradiological factors that predicted the unfavorable outcome, we used logistic regression to quantify the relationship of outcomes against variables corresponding to demographics (age, sex), clinical symptoms (presence of confusion, limbic encephalitis, amnesia, seizures, psychiatric symptoms) and MRI presentations (Table 4). The results showed that these factors did not have statistical significance.

**Table 3:** Comparison of clinical symptoms and outcomes associations among different MRI presentations

	Brain MRI			
	Normal group (*n* = 23)	Limbic system hyperintensities group (*n* = 43)	Non- limbic system hyperintensities group (*n* = 23)	*p* value
**Clinical symptoms**				
Limbic encephalitis at presentation	3	9	3	0.729
Confusion at presentation	3	6	2	0.895
Amnesia at presentation	9	12	11	0.286
Seizures at presentation	1	7	3	0.351
Psychiatric symptoms at presentation	7	9	4	0.812
**Clinical outcomes**				
Death	2	5	3	0.612
Partial recovery	9	24	12	0.538
Return to baseline	12	14	8	0.427

*Abbreviations*: *MRI* Magnetic resonance imaging

**Table 4:** Logistic regression predicting unfavorable outcome

	OR (95% CI)	z value	*p* value
Sex	0.892(0.362–2.196)	−0.249	0.803
Age (years)	0.628(0.215–1.830)	−0.853	0.394
**Clinical symptoms**			
Limbic encephalitis at presentation	1.289(0.400–4.155)	0.425	0.671
Confusion at presentation	1.759(0.433–7.149)	0.789	0.430
Amnesia at presentation	0.835(0.351–2.071)	−0.352	0.725
Seizures at presentation	1.094(0.295–4.055)	0.134	0.893
Psychiatric symptoms at presentation	0.694(0.253–1.904)	−0.709	0.479
Tumor identified	1.901(0.789–4.538)	1.431	0.152
**Brain MRI**			
Normal	0.458(0.175–1.203)	−1.585	0.113
Limbic system hyperintensities	1.539(0.672–3.781)	1.057	0.219
Non- limbic system hyperintensities	1.219(0.453–3.280)	0.392	0.695

*Abbreviations*: *MRI* Magnetic resonance imaging

## Discussion

We summarize local experience with 4 patients and findings from a systematic review of reported anti-AMPAR encephalitis cases. Our findings highlight the marked variability in clinical symptoms, brain MRI manifestations, and clinical outcomes observed in patients with anti-AMPAR encephalitis. Furthermore, neuroradiological abnormalities are not confined to the limbic system; they may extend to atypical sites, including the basal ganglia, cerebellum, and cerebral cortical regions, present as diffuse hyperintensities, or even be absent (i.e., normal brain MRI). Additionally, Fisher’s exact test showed that there was non-significant difference in the clinical symptoms and outcomes among different MRI presentations. Using logistic regression analysis, no significant associations were identified between clinical symptoms, brain MRI findings, disease-associated malignancies, and unfavorable clinical outcomes. A prior study [16] demonstrated that early recognition and prompt initiation of treatment can improve outcomes in this potentially life-threatening disorder. Together these findings may be applied to improve recognition of patients with possible AMPAR, facilitating earlier therapeutic intervention with the objective of improving long-term clinical outcomes.

Based on our case and cases reported in the literature, we observed that the clinical manifestations—including behavioral, cognitive, motor, and sensory symptoms—and clinical outcomes of patients with anti-AMPAR encephalitis exhibit marked variability. We observed that the onset phenotype was limbic encephalitis in 50.6% (45 of 89) of cases. It is well established that cognitive dysfunction constitutes a common manifestation of autoimmune encephalitis, and a growing body of evidence supports an autoimmune pathogenesis underlying cognitive impairment even in the absence of limbic encephalitis [13, 16, 17].

Recent studies have indicated that in younger patients, the presence of confusion at onset is associated with a more favorable prognosis; conversely, the presence of confusion at onset correlates with a poor prognosis during follow-up [18–20]. Previous research has noted a high prevalence of neoplasms in anti-AMPAR encephalitis, with the incidence of paraneoplastic syndrome ranging from 60% to 70% [13, 21, 22]. More recent case series and case reports have also documented a lower frequency of malignancy compared with those in earlier literature, which may be attributed to differences in sample size and follow-up duration. In our retrospective study, malignancy was identified in 40 out of 89 patients (44.9%) with known cancer status. Tsubasa et al. [23] proposed a hypothesis that specific tumor antigens may trigger the production of AMPAR antibodies, thereby contributing to the pathogenesis of anti-AMPAR encephalitis. The review of previously reported cases with tumors suggested that the presence of concurrent paraneoplastic autoimmunity constituted a key prognostic factor for unfavorable outcomes [3, 8, 9, 24, 25]. These findings suggest the necessity of extensive screening for malignancy in patients with psychiatric symptoms.

Brain MRI is considered as a sensitive but not specific diagnostic tool for anti-AMPAR encephalitis [11]. Patients with anti-AMPAR encephalitis most commonly exhibit imaging findings consistent with limbic encephalitis, characterized by bilateral or unilateral T2-FLAIR hyperintensities in the medial temporal lobes [26]. According to the systemic review, brain MRI was frequently abnormal with a stereotyped topography, including a clear predilection for bilateral temporal lobes. Considering the broad distribution of GluR2 AMPAR in the CNS, it is likely that the clinical involvement of anti-AMPAR encephalitis is not restricted to the limbic system and may spread to unexpected sites, like basal ganglia, cerebellum, cerebral cortex regions, and even diffuse hyperintensities. Elamin et al. [27] reported a case of anti-AMPAR encephalitis presenting with altered mental status, while brain magnetic resonance imaging showed no limbic system involvement but posterior cerebral cortex and subcortical white matter involvement. Quaranta et al. [28] reported a case of anti-AMPAR encephalitis complicated with Turner’s syndrome, which started with anxiety, tension, speech rigidity, and other altered mental status and cognitive decline, followed by rapid bidirectional affective disorder. Since the brain MRI and EEG were normal, doctors have been treating the patient with antipsychotic drugs. Anti-AMPAR encephalitis was not diagnosed until six years later. Although MRI findings can confirm the clinical diagnosis of LE, it should be noted that patients with anti-AMPAR encephalitis may have completely normal brain MRI [29]. Our study demonstrated about 74.2% (66 of 89) patients showed abnormalities in brain MRI, with limbic system hyperintensities in 43 patients (Table 2). These findings are consistent with the previously published data. Furthermore, Zhang et al. [26] characterized the cortical ribbon sign in anti-AMPAR encephalitis—an imaging finding that closely mimics that of Creutzfeldt-Jakob disease. On MRI (particularly diffusion-weighted imaging, DWI), this sign manifests as a band of abnormally increased signal intensity traversing the cerebral cortex, conforming to the gray-white matter junction and exhibiting a morphological pattern analogous to a cortical “ribbon”. Pathophysiologically, the emergence of the cortical ribbon sign in anti-AMPAR encephalitis is hypothesized to be secondary to cytotoxic edema within the cortical lesions. This underlying pathological process offers a plausible mechanistic explanation for the distinct radiological hallmark observed in this subset of autoimmune encephalitides. Wei et al. [10] reported a patient with anti-AMPAR encephalitis who developed progressive hippocampal atrophy and increased T2-FLAIR signal intensity; such progressive hippocampal sclerosis was correlated with short-term memory deficits and long-term cognitive impairments during follow-up [29]. Additionally, magnetic resonance spectroscopy (MRS) revealed a decrease in the NAA peak, indicating neuronal cell damage, and an increase in the Cho peak, suggesting cellular inflammatory edema, cellular membrane disintegration, demyelination of white matter, and heightened cellular metabolic function.

EEG usually shows focal or generalized slowing, epileptiform activity, and periodic lateralized epileptiform discharges and is helpful in excluding other causes of non-convulsive seizures and encephalitis [15]. However, it was reported in the literature that EEG was less sensitive than brain MRI, and only 44% of patients had EEG abnormalities [28]. CSF studies often show predominant lymphocytic pleocytosis and the presence of AMPAR antibodies. The diagnosis requires the detection of cerebrospinal fluid and serum AMPAR antibody. Once the antibody is detected, the diagnosis can be made [30].

Treatment of anti-AMPAR encephalitis includes first-line therapies (IVIG, steroids, and plasmapheresis) and second-line therapies (rituximab and immunosuppressants, etc.) and therapy usually combines two or more treatment options [29]. Early treatment and no admission to an intensive care unit were identified as predictors of good outcome [31, 32]. More than half of the patient’s memory deficits did not completely recover despite the administration of immunotherapy. The persistence of the neurologic deficits and eventual fatality may occur depending on the severity of the disease and complications of other organs [33]. During the follow-up, residual memory deficit was the most common symptom by reviewing literature, and most of the patients would improve gradually after reasonable immunotherapy [34].

In this study, Fisher’s exact test showed that there was non-significant difference in the clinical symptoms among different MRI presentations. By using logistic regression, we found no significant relationships between clinical symptoms, Brain MRI, disease-associated malignancy and unfavorable outcome. We supposed that the range of hyperintensity on MRI may not be directly associated with patients’ clinical symptoms and outcomes. Additionally, in two of our cases, radiologic improvement did not parallel clinical recovery. It may also be related to the small sample size limiting the statistical power and hindering a firm conclusion, or to the multicollinearity among the independent variables. Larger prospective studies are needed to clarify imaging-clinical correlations. Further, Hoftberger et al. [12] reported clinical features and treatment profile of 22 subjects and found that patients with or without tumor did not show any difference in the clinical outcomes but those patients with tumor and associated autoimmune antibodies had poor prognosis. Gresa-Arribas et al. [35] found that NMDAR-Ab titers were higher in patients with poor outcome or teratoma than in patients with good outcome or no tumor, and the titer change in CSF was more closely related to relapses than was that in serum. Yang et al. [36] reported that additional neuronal antibodies (CRMP5) were detected in the patient, which may be associated with a poor prognosis. Further studies are needed to decipher more risk factors related to clinical outcomes.

However, the limitations of the study are obvious. First, the small sample size limits the statistical power, therefore hindering a firm conclusion. Second, the search strategy is not powerful enough. Single-database search may involve potential biases and a systematic search using the key terms may miss the relevant cases indexed by alternative terms. Third, only patients clinically suspicious of encephalitis were tested for anti-neuronal antibodies. Fourth, the limited follow-up period may affect the assessment of the results. Better understandings of this disease, rely on deeper investigations into the pathological mechanisms and the accumulation of patient cohorts.

## Conclusions

We presented four typical cases of anti-AMPAR encephalitis; in addition, we summarized the clinical and neuroradiological features of the patients with anti-AMPAR encephalitis, while discussing the relationship between neuroradiological presentations and outcomes. The current findings, taken together with those of previous studies, have several practical implications. First, it is likely that the clinical involvement of anti-AMPAR encephalitis is not restricted to the limbic system and may spread to unexpected sites, like basal ganglia, cerebellum, cerebral cortex, and even diffuse hyperintensities, and it should be noted that patients with anti-AMPAR encephalitis may have completely normal brain MRI. Second, the range of hyperintensity on MRI may not be directly associated with patients’ clinical symptoms and outcomes. Third, for the expanding clinical profile of encephalitis associated with antibodies against AMPAR, careful databasing of new cases will facilitate more definitive studies in the future.

## Data Availability

All data contained within the article.

## Abbreviations

AMPAR: α-amino-3-hydroxy-5-methyl-4-isoxazole-propionic acid receptor
CSF: Cerebral spinal fluid
EEG: Electroencephalogram
FLAIR: Fluid-attenuated inversion recovery
IVIG: Intravenous injection of immunoglobulin
MRI: Magnetic resonance imaging
MRS: magnetic resonance spectroscopy
